# CRISPR/Cas9 Delivery Systems to Enhance Gene Editing Efficiency

**DOI:** 10.3390/ijms26094420

**Published:** 2025-05-06

**Authors:** Ana Seijas, Diego Cora, Mercedes Novo, Wajih Al-Soufi, Laura Sánchez, Álvaro J. Arana

**Affiliations:** 1 Departamento de Zooloxía, Xenética e Antropoloxía Física, Facultade de Veterinaria, Universidade de Santiago de Compostela, 27002 Lugo, Spain; 2 Departamento de Química Física, Facultade de Ciencias, Campus Terra, Universidade de Santiago de Compostela, 27002 Lugo, Spain

**Keywords:** CRISPR, Cas9, gene editing, delivery systems, non-viral vectors, LNPs, Cas9 aggregation, nanoparticle encapsulation, physical delivery

## Abstract

CRISPR/Cas9 has revolutionized genome editing by enabling precise and efficient genetic modifications across multiple biological systems. Despite its growing therapeutic potential, key challenges remain in mitigating off-target effects, minimizing immunogenicity, and improving the delivery of CRISPR components into target cells. This review provides an integrated analysis of physical, viral, and non-viral delivery systems, highlighting recent advances in the use of lipid nanoparticles, polymeric carriers, and hybrid platforms. We also examine an often overlooked factor: the aggregation behavior of the Cas9 protein, which may interfere with cellular uptake, the encapsulation efficiency, and nuclear localization. By comparing delivery platforms and their reported editing outcomes, we identify critical physicochemical parameters that influence therapeutic success. Finally, we propose standardized methods to assess Cas9 encapsulation and aggregation and discuss translational barriers such as manufacturing scalability and regulatory requirements. These insights aim to guide the development of safer and more effective CRISPR/Cas9-based therapies.

## 1. Introduction

The CRISPR/Cas9 (clustered regulatory interspaced short palindromic repeats/CRISPR-associated 9) system has emerged as a revolutionary and powerful tool in the scientific community due to its gene editing potential in multiple cell types and organisms, holding great potential for therapeutic applications [1]. The combination of guide RNA (gRNA) and the Cas9 enzyme to form a ribonucleoprotein (RNP) complex is crucial to make cuts at specific locations in DNA sequences, making it a promising tool in molecular biology and therapeutic research [2]. However, CRISPR/Cas9 clinical applications present significant challenges, particularly regarding the safe and efficient delivery of its components into target cells with high specificity [3]. Therefore, research still needs to overcome significant obstacles regarding immune reactions, off-target effects, and suboptimal delivery efficiency.

The gene editing efficiency depends strongly on the delivery of CRISPR/Cas9 components into the cell nucleus. As a result, different delivery methods to introduce CRISPR/Cas9 into cells and organisms, such as viral vectors, lipid nanoparticles (LNPs), exosomes, or physically derived technologies, have been extensively studied. Consequently, numerous reviews have already been published focusing not only on the CRISPR/Cas9 system but also on different types of delivery methods [4,5,6,7,8]. A recent clinical milestone that illustrates the importance of these efforts is the FDA approval of CASGEVY^TM^ (CTX001), the first CRISPR/Cas9-based gene therapy, for the treatment of sickle cell disease and transfusion-dependent β-thalassemia [9,10]. Although this therapy uses the ex vivo electroporation of autologous stem cells, it highlights the translational promise of CRISPR/Cas9 and the urgent need to develop efficient and safe delivery platforms for broader clinical applications.

Recent studies have demonstrated the therapeutic potential of in vivo genome editing using non-viral delivery systems. Chen et al. developed a thermostable Cas9 variant (iGeoCas9) formulated in lipid nanoparticles, achieving efficient nuclease-mediated editing in the liver and lung. In parallel, Bzhilyanskaya et al. reported successful PAMless base editing in hematopoietic stem cells, supporting the feasibility of precise systemic genome editing. These advances highlight the translational relevance of CRISPR technologies. Furthermore, studies on CRISPR-associated immunogenicity suggest that pre-existing immunity to Cas9, while present in the serum, may pose a reduced risk in immune-privileged organs such as the eye, although systemic delivery remains a concern [11,12].

Another important and often overlooked factor that can influence the genome editing efficiency is protein aggregation [13]. Protein aggregation consists of the abnormal clustering of proteins, creating products that can range from small dimers to large assemblies, which tend to be insoluble, and can occur under both normal physiological conditions and in response to stress, such as from temperature fluctuations or pH adjustments, as has been previously studied [14,15]. In the context of gene therapy, Cas9 aggregation can lead to the formation of particles which exceed the optimal size range for its delivery into cells, compromising the delivery efficiency [13].

This review was conducted using PubMed, Scopus, Web of Science, and Google Scholar, focusing on CRISPR/Cas9 delivery, gene therapy vectors, protein stability, and nanoparticle-based approaches. During this process, we noticed that Cas9 aggregation, although mentioned in some studies, had not been fully studied and that providing explanations on why it occurs and how it affects delivery had been avoided. Therefore, this review focuses on cargo types and delivery vehicle methodologies including physical, viral, and non-viral approaches, highlighting innovative delivery systems, their strengths and weaknesses, and their efficiency in genome editing. Moreover, we also briefly remark on Cas9 aggregation’s implications for gene editing and the delivery efficiency.

## 2. CRISPR/Cas9 Delivery Systems

CRISPR/Cas9 gene editing success relies heavily on delivery systems, as these directly influence the transfection efficiency, target specificity, and therapeutic potential [16]. Since the CRISPR/Cas9 complex must function in the nuclear genome, its components need to be efficiently delivered into the nucleus, which requires it to overcome tissue and cell membrane barriers while facing challenges due to its considerable molecular weight and its poor stability [8].

Previous reviews [4,8,17] have already mentioned that each delivery strategy comprises two key components: the cargo and the vehicle. The cargo refers to what components are delivered into the cell to enable gene editing, and it typically includes CRISPR elements such as plasmid DNA (pDNA) encoding the Cas9 protein and gRNA; messenger RNA (mRNA) for Cas9 protein translation, paired with gRNA; or pre-assembled Cas9/gRNA RNP complexes. The vehicle’s main function is to transport cargoes and introduce them into target cells using different methodologies [8].

**Figure 1:**
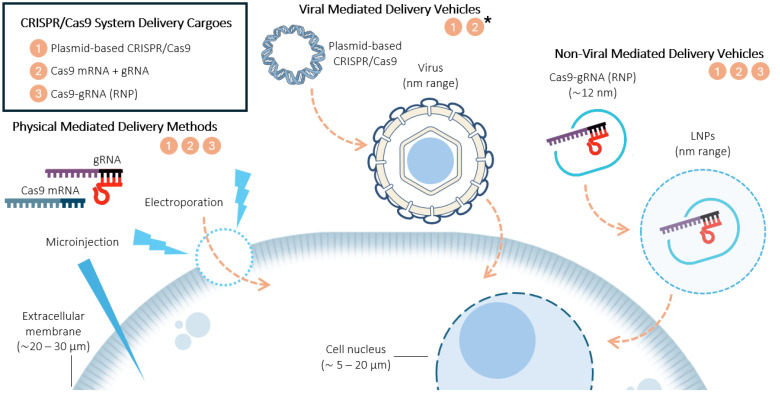
Scheme of all three types of CRISPR/Cas9 system delivery cargoes: plasmid-based CRISPR/Cas9 and Cas9 mRNA coupled with gRNA and RNP complexes. In addition, a representation of the different types of delivery vehicles is included: physically mediated delivery methods, virally mediated delivery vehicles, and non-virally mediated delivery vehicles. Each delivery vehicle is associated with specific cargo types, as indicated by the corresponding numbers below each title. Viral vector cargoes (*) are included in the plasmid-based and mRNA categories to facilitate comprehension, as they deliver DNA or RNA encoding Cas9 and gRNA as part of their genome. The possible cargoes for each delivery vehicle are denoted with their corresponding numbers under each title. Illustrations were created with https://bioart.niaid.nih.gov (accessed on 5 April 2025).

Due to their importance and innumerable applications, recent research has focused on CRISPR/Cas9 delivery systems, and a classification of both CRISPR/Cas9 system delivery cargoes and different types of delivery vehicles (Figure 1) will be discussed hereafter, focusing on their strengths, weaknesses, and editing efficiency.

### 2.1. CRISPR/Cas9 System Delivery Cargoes

There are three main forms of cargoes in delivery systems regarding CRISPR/Cas9 technology. Their associated advantages and drawbacks are discussed below.

- Plasmid-based CRISPR/Cas9. This system is widely used due to its simplicity and low-cost manipulation [18]. The moderate toxicity reported in certain cell lines could limit its application as the optimal lipid composition may be different [19]. In addition, both the large size of Cas9 and the need for nuclear entry limit its genome editing efficiency [8,18]. gRNA can be encoded within the plasmid alongside Cas9 or introduced separately as a synthetic gRNA for more precise control over the editing efficiency [20]. Viral vector cargoes might also be included in this category, as they typically deliver DNA or RNA encoding both Cas9 and gRNA, rather than introducing them separately, leading to either genome integration or transient or episomal expression [7].
- Cas9 mRNA coupled with gRNA. This method offers fast and low-toxicity genome editing, making it ideal for sensitive cells. Liu et al. [21] clearly demonstrated its biocompatibility and high genome editing efficacy using bioreducible LNPs for the simultaneous delivery of Cas9 mRNA and gRNA. This system decreases off-target editing events, making it suitable for the transient expression of Cas9 [18]. The gRNA can either be co-transcribed within the Cas9 mRNA or delivered separately as an independent molecule to optimize stability and efficiency. Viral vector cargoes could also be included in this category when delivering RNA encoding Cas9 and gRNA without genome integration.
- RNP complexes. RNPs are composed of a Cas9 protein and gRNA, and they offer the highest gene editing efficiency and specificity [20]. Wei et al. demonstrated that lipid nanoparticles encapsulating RNPs exhibited tissue-specific gene editing in mice’s lungs and liver [22]. Moreover, this system also minimizes off-target effects and toxicity [18,22].

Thus, while the selection of a CRISPR/Cas9 cargo influences the gene editing efficiency and specificity, its successful application depends on using the appropriate delivery method. Effective genome editing requires not only selecting the most suitable cargo but also ensuring its efficient transport into target cells (Figure 1). Each delivery strategy must overcome cellular barriers, optimize the uptake, and achieve precise localization to maximize the editing efficiency.

Delivery strategies, along with their specific applications and limitations, are discussed in detail in the following sections.

### 2.2. Types of Delivery Vehicles

There are three primary strategies for delivering CRISPR/Cas9 components into cells (Figure 1), each influencing the genome editing efficiency based on its mechanism of action and delivery vehicle (Table 1). Physical delivery methods facilitate plasmid transfer and the transportation of mRNA or RNPs with high efficiency, though their application may be limited by cell viability and specificity [21]. Viral vectors vary in their genetic material and they transport either DNA or RNA depending on their genomic structure, influencing whether they mediate transient expression or integrate into the host genome, while also posing size constraints and potential immunogenic risks [7,23]. Non-viral systems offer safer alternatives by encapsulating plasmids, mRNA, or pre-formed Cas9:gRNA RNPs, though their efficiency relies on uptake mechanisms and overcoming biological barriers [5,9,17,18,19]. Since effective encapsulation and delivery depend on electrostatic interactions, the opposite charges on Cas9 (positive) and oligonucleotides or Cas9:gRNA complexes (negative) must be balanced with well-designed carriers. Lipid-based systems, for example, use cationic lipids to form stable lipoplexes that enhance the payload protection, cellular uptake, and endosomal escape, ultimately improving the genome editing efficiency [21,22].

**Table 1:** This table summarizes some examples of the reviewed delivery methods focusing on the editing efficiency and some details of the experimental procedures. Note that in some references, no quantitative data on the editing efficiency were reported.

Delivery Method	Details	Editing Efficiency	Ref.
Physically mediated	Microinjection used to insert green fluorescent protein into HepG2 cells	Around 40%	[24]
	Electroporation in mouse zygotes	Reported as being highly efficient (no quantitative data provided)	[25]
	Electroporation of Cas9 RNP in HSPCs (CASGEVY^TM^—ex vivo)	Up to 90% indels in BCL11A enhancer	[9]
	Electroporation of Cas9 in somatic cells (in vitro)	High editing efficiency reported (no quantitative data provided)	[26]
Virally mediated vehicles	Use of AAVs to specifically edit cell lines in mouse nervous system	Reported as being efficient (no quantitative data provided)	[27]
	Use of engineered AAVs with capsid modifications to enhance transduction	Improved editing reported in muscle and neural tissues (no quantitative data provided)	[28,29]
	Use of lentiviral vectors optimized for stable genome editing in hematopoietic and liver cells	High editing efficiency and long-term expression reported (no quantitative data provided)	[30]
Non-virally mediated vehicles	Bioreducible LNPs	Up to 90% in cultured cells and up to 80% in vivo	[21]
	LNPs used to deliver RNPs into cells and edit tissues such as muscles, brain, liver, and lungs	High editing efficiency reported (no quantitative data provided)	[22]
	Bioreducible LNPs with negative charge used in mammalian cells and rodent brain	Approximately 70%	[31]
	Nanoparticles with polyglutamic acid used in different types of T cells	Knockin efficiency of up to >50%	[13]
	LNPs delivering iGeoCas9 RNP (engineered thermostable Cas9) to liver and lung in vivo (mouse)	16–37% in liver and 19% in lung	[32]
	Polyplex micelles (PEG–PLL) used for Cas9 mRNA/sgRNA delivery to mouse brain (intraparenchymal)	~20%	[33]
	C14–PEI micelleplexes (non-coated) used for Cas9 mRNA/sgRNA delivery to KRAS-mutant lung cells	~60% (T7EI), 48.5% (ddPCR)	[34]
	PEG–PLE/C14–PEI micelleplexes used for Cas9 mRNA/sgRNA delivery to KRAS-mutant lung cells	Up to 69%	[35]
	Mini enveloped delivery vehicles (EDVs)	Up to ~85% indels in HeLa and U2OS cells	[26]
	Gold nanoparticles used to correct mutations of Duchenne muscular dystrophy in different cell types	5.4% of gene edited to wild type	[36]
	Nanoscale ZIFs	37% reduction in gene expression	[37]

Note: The editing efficiencies are reported as described in the original studies. When quantitative values were not provided, qualitative descriptors (e.g., “high”, “efficient”) have been retained.

#### 2.2.1. Physically Mediated Delivery Methods

Physical delivery methods apply physical forces to facilitate the intracellular uptake of CRISPR/Cas9 components through host cellular and nuclear membrane disruption [5]. Physical methods including microinjection, electroporation, hydrodynamic injection, and sonoporation play a critical role in introducing CRISPR/Cas9 components into cells, offering high precision and versatility across various applications.

Microinjection allows for the direct delivery of RNPs, plasmid DNA, or mRNA into the cytoplasm or nucleus, making it highly effective for germline editing in model organisms such as zebrafish and mice [24,38,39]. However, its applications in in vivo gene therapy are highly limited because it is labor-intensive, impractical for large-scale systemic administration, and with an outcome highly dependent on the operator. In mammals, microinjection is typically performed in zygotes, requiring the extraction of oocytes, in vitro fertilization, and subsequent embryo implantation. This makes it a feasible approach only for germline editing and not for treating genetic diseases in already born individuals, particularly in adults, where systemic delivery methods are required to reach the affected tissues efficiently [24,39].

Electroporation, which uses electric fields to create transient pores in the cell membrane to allow for the uptake of macromolecules such as CRISPR/Cas9 components, has demonstrated high transfection efficiency in induced pluripotent stem cells (iPSCs), T cells, and zygotes among others [9,25]. While electroporation is widely used in ex vivo cell therapies, its direct in vivo applications are constrained by potential cellular stress and tissue damage, with low efficiency and viability. Moreover, its effects are limited to the electroporated area, making it unsuitable for therapies requiring widespread gene editing across multiple tissues. In addition, it is a costly method, making its clinical applications limited [23]. However, advances in microscale electroporation systems have improved the reproducibility, increasing the delivery efficiency.

Other physical methods, such as hydrodynamic injection and sonoporation, offer alternative approaches. Hydrodynamic injection involves rapidly injecting large volumes of CRISPR components into the bloodstream with high-pressure application, achieving high delivery efficiency for a mouse liver through tail vein injection [40,41]. Meanwhile, sonoporation uses low-level ultrasound waves to alter the plasma membrane, allowing for the introduction of editing material into cells, although low gene expression levels are its main drawback [42].

Despite their advantages, physical methods often face limitations in terms of scalability, tissue specificity, and potential cellular damage; therefore, several approaches have been developed to enhance the CRISPR/Cas9 delivery efficiency and biocompatibility in research and therapeutic studies.

A recent clinical milestone illustrating an application of physical delivery methods is the approval of CASGEVY^TM^ (CTX001) by the FDA. This autologous ex vivo gene editing therapy uses electroporation to introduce Cas9 RNPs into hematopoietic stem and progenitor cells (HSPCs), targeting the BCL11A erythroid enhancer to induce fetal hemoglobin production. The edited cells are then reinfused into the patient [9,10]. Despite its clinical success, this approach is restricted to hematologic disorders and highly specialized facilities due to the invasive nature of cell isolation, complex manufacturing processes, and the inability to directly target most tissues. These constraints underscore the need for compatible and scalable in vivo delivery systems, especially non-viral platforms suitable for systemic administration.

#### 2.2.2. Virally Mediated Delivery Vehicles

Virally engineered vectors such as lentiviruses, adenoviruses (AVs), and adeno-associated viruses (AAVs) have been extensively used for delivering CRISPR/Cas9 components due to their high transduction efficiency and ability to transfer genetic material into a wide range of cell types [7,43,44,45,46,47]. AAVs, in particular, have become one of the most widely used vectors in gene therapy because of their non-pathogenic nature and their capacity to transduce both dividing and non-dividing cells with high efficiency [29,43,45]. Lentiviral vectors, by contrast, offer the advantage of stable genome integration, making them highly suitable for the ex vivo editing of proliferative cells, such as hematopoietic stem cells [30,43,44]. Depending on the vector type, CRISPR/Cas9 components can be expressed either transiently (as in AAV or AV systems) or stabilized through genomic integration (as in lentiviral vectors), with different safety and efficiency implications.

Despite their broad utility, viral vectors present several limitations, of which the most relevant is the immune response against viral capsid proteins, which can reduce the transduction efficiency [48,49,50]. Using vectors such as lentiviruses and retroviruses also poses risks of off-target genome integration and insertional mutagenesis, potentially leading to genotoxic effects [44,48,51]. Additionally, the limited cargo capacity of AAV vectors (around 4.7 kb) restricts their ability to deliver larger CRISPR/Cas9 systems, especially when including regulatory elements or several gRNAs [27,43,46,52].

To overcome these challenges, viral vectors have been the focus of extensive studies. In AAVs, capsid modifications have been introduced to improve tissue selectivity and avoid immune detection, while lentiviral vectors have been optimized to enhance nuclear entry and ensure long-term expression in specific cell types [28,29,30,43]. More recently, alternative viral platforms such as bacteriophage-derived systems have shown promising results in improving specificity and reducing immunogenicity, achieving efficiencies comparable to those of AAVs in certain models [43,53]. Efficient methods of viral delivery, particularly AAV-based systems, have been demonstrated in preclinical models, showing efficient gene editing in the neural tissues of mice (Table 1) [27,54].

While non-viral system development has opened the scope to safer and more flexible alternatives, viral vectors continue to be the most widely used and clinically validated method for CRISPR/Cas9 delivery. Their unmatched transduction efficiency, versatility, and proven performance in both preclinical and clinical settings underscore their central role in genome editing today, especially in applications where long-term expression or in vivo delivery is essential [43,46,55].

#### 2.2.3. Non-Virally Mediated Delivery Vehicles

Non-viral delivery systems have emerged as a promising alternative to viral vectors, offering increased safety and versatility in delivering CRISPR/Cas9 components. Unlike viral systems, non-viral vehicles pose lower risks of immunogenicity and adverse effects associated with genomic integration [56].

Among these, LNPs are particularly noteworthy for their ability to encapsulate and protect CRISPR components, enhancing the cellular uptake and stability against enzymatic degradation [21]. LNPs’ structure and composition facilitate fusion with the cell membrane, allowing for the intracellular delivery of CRISPR/Cas9 components to target cells [57]. LNPs have proven to be especially effective in delivering mRNA and RNP complexes, achieving efficiencies of up to 97% in several studies [5,16,31]. Recent innovations in LNP design have significantly improved their biocompatibility, encapsulation ability, stability, and targeting specificity; combining LNPs with bioreducible lipid-like materials such as cholesterol, 1,2-dioleoyl-*sn*-glycero-3-phosphorylethanolamine (DOPE), anionic polymers, and polyethylene glycol (PEG)-derived lipids has demonstrated up to 90% transfection efficiency in vitro and in vivo [3,13,19,31,58]. Supporting their clinical relevance, Chen et al. demonstrated that LNP-encapsulated cytosine base editors enabled the successful in vivo correction of a metabolic liver disease, providing functional rescue in mouse models [32].

Recent breakthroughs have expanded the versatility of lipid-based delivery systems through the development of Selective Organ Targeting (SORT) LNPs. This strategy introduces a fifth lipid component that modulates biodistribution, enabling the precise redirection of CRISPR/Cas9 cargoes to extrahepatic tissues such as the lung, spleen, or heart. Wang et al. formulated SORT LNPs incorporating DOTAP or 18PA to selectively target the lung or spleen, respectively, and demonstrated the efficient systemic delivery of mRNA and Cas9 RNPs in mice [59]. In parallel, Su et al. reformulated conventional LNPs by eliminating cholesterol and phospholipids, introducing specialized lipids that enhanced pulmonary targeting while reducing hepatic accumulation [60]. These innovations offer a modular approach to improving the specificity and performance of lipid-mediated genome editing.

Building on these developments, Guzman Gonzalez et al. addressed a critical limitation of LNPs for in vivo delivery: their preferential accumulation in hepatic tissues, which restricts their utility for systemic and extrahepatic gene delivery [61]. An innovative alternative was proposed through the development of cell-penetrating peptides capable of stably self-assembling with CRISPR/Cas9 RNA into stable Peptide-Based Nanoparticles called ADGNs, which encapsulate CRISPR components and efficiently target cells overexpressing laminin receptors, with laminin being a characteristic cell surface glycoprotein upregulated in most cancer cells. Their findings demonstrated significant improvements in the cellular uptake, stability, and gene editing efficiency both in vitro and in vivo, avoiding preferential accumulation in hepatic tissues and allowing for extrahepatic delivery, specifically to the lungs of mice. This work highlights ADGNs’ potential to broaden the scope of non-viral delivery systems beyond the hepatic scope, opening new avenues for the use of CRISPR-based therapies in oncology and to treat other systemic diseases.

Modified natural polymer carriers, such as chitosan, have also demonstrated significant potential in CRISPR/Cas9 delivery due to their ideal biodegradability and high biocompatibility. For instance, carboxymethyl chitosan combined with AS1411 ligands achieved over 90% delivery efficiency for plasmid DNA, specifically targeting cyclin-dependent kinase 11 (CDK11) expression in cancer cells [62]. Similarly, DNA nanoclews, yarn-like DNA nanoparticles synthesized through rolling circle amplification, offered a gene editing approach in in vitro human cell trials [63]. Moreover, modified polyethylenimine (PEI) polymers have enabled the selective release of CRISPR components, enhancing therapeutic efficacy and safety despite the fact that high-molecular-weight PEI can induce cytotoxicity [5,17,55].

As an alternative example, hybrid systems combining polymers with inorganic nanoparticles, such as gold nanoparticles, have further expanded the delivery possibilities, offering synergistic benefits. Arginine-functionalized gold nanoparticles provide a biocompatible and non-toxic platform for delivery applications [64]. Moreover, gold nanoparticles have already been shown to deliver Cas9 and DNA into a wide variety of cell types, as well as demonstrated therapeutic potential by restoring dystrophin expression in Duchenne muscular dystrophy in mice [36]. Zeolitic imidazolate frameworks (ZIFs) and silica nanoparticles offer additional advantages, providing the high-capacity encapsulation and protection of CRISPR components and achieving effective gene editing in cancer models [17,37,57,65].

Another promising non-viral delivery strategy involves mini enveloped delivery vehicles (EDVs). These lipid-based particles are derived from retrovirus-like structures and incorporate components such as Gag or VSV-G proteins to facilitate cellular uptake. However, unlike classical viral vectors, EDVs do not deliver genetic material nor integrate into the host genome, functioning instead through the transient delivery of Cas9 RNP complexes. Recent findings showed that EDVs can achieve editing efficiencies of up to ~85% in human somatic cell lines such as HeLa and U2OS, with faster editing kinetics compared to electroporation [26]. Although limitations in the stability and intracellular distribution of Cas9 have been reported (see Section 4), EDVs’ non-integrative and virus-free mechanism supports their classification as a non-viral delivery platform.

Among the recent advances in polymer-based vectors, polyplex micelles (PMs) have gained attention for their tunable properties and co-delivery potential. Abbasi et al. developed PEG–PLL polyplex micelles that enabled the co-delivery of Cas9 mRNA and sgRNA to neurons, astrocytes, and microglia via intraparenchymal administration, achieving ~20% editing efficiency in the mouse brain [33]. Separately, Chen et al. reported that C14-PEI micelleplexes achieved ~60% editing efficiency according to T7EI and 48.5% according to droplet digital PCR (ddPCR) in KRAS-mutant lung cancer cells, confirming efficient complexation and cellular uptake [34,35]. Building on this, PEG–PLE-coated C14–PEI micelleplexes further enhanced colloidal stability, reduced cytotoxicity, and improved endosomal escape, reaching up to 69% indels in vitro and showing improved performance in hard-to-target tissues. These findings collectively support the potential of PMs as a modular and efficient platform for systemic CRISPR/Cas9 delivery.

Emerging technologies continue to address traditional delivery systems’ limitations by integrating advanced materials and hybrid platforms. The hybridization of exosomes and liposomes demonstrated high specificity and the efficient delivery of CRISPR/Cas9 using plasmids in both in vitro and in vivo assays [66]. Furthermore, novel developments in gene editing techniques, such as base editing and prime editing, have enabled specific point mutations without generating double-strand DNA breaks, boosting CRISPR/Cas9’s therapeutic potential [43]. More recently, PAMless base editors, which do not require a specific protospacer-adjacent motif (PAM) near the target site, have expanded the range of editable genomic loci, overcoming sequence constraints that limit conventional editors [67]. Bzhilyanskaya et al. demonstrated the efficient delivery of such editors into hematopoietic stem cells, further supporting their clinical potential. Together, these developments underscore the adaptability of polymeric, inorganic, and hybrid delivery systems in overcoming the current limitations, thereby improving the safety, efficiency, and specificity of CRISPR/Cas9-based therapies.

## 3. Aggregation Behavior of Cas9 Protein

In the process of the creation of this review, we stumbled upon some information regarding the solubility and aggregation behavior of Cas9. Some authors suggest that the buffer composition, pH, and presence of gRNA significantly influence this behavior. Despite this, the extent to which these factors impact the gene editing efficiency remains unclear. We hypothesized that Cas9’s aggregation, or the presence of gRNAs, could affect its internalization into cells and delivery systems and ultimately worsen the CRISPR/Cas9 gene editing efficiency [13,22,68,69,70,71]. However, there is currently no direct evidence on how aggregation might alter the performance of different delivery methods, making this a critical but unresolved question in the field.

Manzano et al. observed Cas9 aggregation, attributing it to shear-induced processes during stirring, particularly at pH 8 near its isoelectric point [70]. They determined the effective size of RNP complexes by dynamic light scattering (DLS) in 20 mM Tris and in 300 mM NaCl (pH 8.0), obtaining monodisperse distributions in both conditions with mean diameters of 10.3 ± 1.5 nm and 12.2 ± 1.5 nm, respectively. Nguyen et al. reported a Cas9 size range of 10–15 nm in PBS buffer, which aligns with Manzano et al.’s results [13]. However, this value increased by up to 200 nm upon the addition of gRNA, suggesting aggregate formation [70]. They also noted, however, that Cas9 was relatively insoluble and that the solubility increased with the amount of gRNA, giving incoherent results. Additionally, Camperi et al. observed small amounts of Cas9 or RNP complex aggregates and identified different types and levels of aggregation in various sources of gRNA materials by size exclusion chromatography instead [71].

Importantly, while protein-based Cas9 RNP complexes are particularly prone to aggregation due to the direct interaction between Cas9 and sgRNA, aggregation-like behavior may also compromise mRNA-based delivery systems when colloidal instability arises [13]. For example, an improper charge balance, polydispersity, or surface interactions in polymeric micelleplexes can result in functional loss or reduced intracellular trafficking, even in the absence of visible aggregates. Studies by Abbasi et al. and Chen et al. support this broader view: PEG–PLL and PEG–PLE coatings were shown to enhance micelle stability, reduce non-specific interactions, and improve the Cas9 mRNA delivery efficiency in both neural and cancer models [33,34,35]. These findings highlight that aggregation control (whether physical or functional) should be a general design principle across delivery modalities.

We propose to investigate this phenomenon using different techniques, such as DLS, as was used in the previously mentioned articles, or fluorescence correlation spectroscopy (FCS), which has been used to detect and analyze early protein aggregation processes by our group before [68,69]. What is more, using these techniques, the size and conformation of the delivery systems, as well as the internalization of Cas9 or other cargoes, can be studied. Understanding these parameters is crucial for optimizing CRISPR-based therapeutic applications, as different aggregation states may affect intracellular processing and activity.

Recent insights from Karp et al. provided direct evidence on the intracellular behavior of electroporated Cas9, revealing punctate cytoplasmic distribution patterns that may result from protein aggregation or entrapment. Using FCS, they determined that a high editing efficiency requires the presence of over 1300 Cas9 RNP complexes per nucleus, suggesting that any form of aggregation or cytoplasmic sequestration could significantly impair functional delivery. Interestingly, these findings align with previous hypotheses that aggregation may reduce nuclear access and gene editing efficiency even when Cas9 is delivered using efficient methods like electroporation [26].

No research has addressed how aggregation affects Cas9 delivery and function, so data are not yet available. Nevertheless, as we will discuss in the next section, there is quantitative evidence on the encapsulation efficiency of Cas9 in different systems, which may offer indirect insights into how aggregation could influence the delivery performance. Aggregation could disrupt encapsulation within lipid nanoparticles or intracellular mobility after electroporation, microinjection, or hydrodynamic injection. It could reduce the editing efficiency or, unexpectedly, provide stability. It could also impact immune recognition and the biodistribution in vivo. Aggregation may alter Cas9’s size and structure, affecting its stability, solubility, function, and consequently, its delivery. If aggregates are too large, they could hinder cellular uptake and reduce bioavailability. In nanoparticles or polymeric delivery systems, excessive aggregation could reduce the encapsulation efficiency. In viral vectors, misfolding could limit the number of active particles. Even inside the cell, aggregation could impair nuclear import and interactions with genomic DNA.

To mitigate these challenges, several strategies have been explored to enhance the Cas9 and RNA stability and prevent aggregation. Polyglutamic acid coatings have demonstrated the ability to stabilize Cas9 RNPs, reducing aggregate formation and improving nanoparticle encapsulation [9]. Additionally, pre-heating gRNA before complex formation has been shown to improve the RNP stability and minimize irreversible aggregation [62].

Additional studies have also highlighted the relevance of structural engineering in mitigating aggregation. For example, Chen et al. developed a thermostable Cas9 variant (iGeoCas9) and demonstrated that its improved solubility facilitated higher encapsulation in lipid nanoparticles and enhanced the in vivo editing outcomes, with up to 37% editing efficiency in the liver and 19% in the lungs. These results strengthen the hypothesis that aggregation resistance contributes to better intracellular trafficking, endosomal escape, and ultimately, editing activity [32].

Together, these findings suggest that aggregation should be considered not only a physicochemical challenge but also a biological barrier to efficient Cas9-based therapies. The future optimization of RNP formulations, protein engineering, and intracellular delivery strategies should explicitly account for aggregation behavior to ensure successful genome editing.

## 4. Encapsulation Efficiency of Cas9 Protein

Cas9 delivery remains one of the main challenges in genome editing due to the molecule’s large size and potential instability. While functional outcomes such as gene editing rates are frequently reported, quantitative assessments of the Cas9 encapsulation efficiency are less common. Recent studies have focused on the encapsulation performance of different delivery vehicles (Table 2). Ponomareva et al. clearly showed the low yield of passive loading strategies, with only ~1% of exosome-like vesicles containing detectable Cas9 protein. In contrast, gold nanoparticle-based systems demonstrated remarkably precise control over Cas9 loading [72]. Therefore, ensuring Cas9 delivery is essential, but a deep understanding of how much protein is effectively carried and retained within the carrier is also vital. Moreover, systems such as the one studied by Li et al. based on glycosylated and cascade-responsive nanoparticles, although specific encapsulation ratios were not provided, achieved highly efficient functional delivery in glioblastoma models, suggesting optimized loading and release under tumor-specific triggers [73].

The particle size and homogeneity are extremely related to the encapsulation outcomes. Systems with narrow size distributions of around 100–200 nm, such as gold-based platforms and optimized LNPs, generally have better intracellular uptake. For instance, Konstantinidou et al. described a system where Cas9 is conjugated to a gold nanoparticle with a hydrodynamic size of ~23 nm, allowing for nuclear localization and gene editing activity without transfection reagents [74]. In contrast, aggregation-prone systems often produce larger and more heterogeneous populations, preventing endosomal escape and biodistribution. While Manzano et al. reported RNP complex sizes of ~10–12 nm under neutral salt buffers, these expanded to 200 nm upon complexation with gRNA, probably due to aggregation [70]. Such shifts not only alter the particle uptake kinetics but also reduce the effective loading capacity per volume and may increase immune recognition.

Recent findings by Karp et al. underscore that the delivery efficiency must be assessed not only according to encapsulation metrics but also according to intracellular trafficking and protein integrity [26]. Their fluorescence-based analysis showed that electroporated Cas9 often accumulated in discrete cytoplasmic foci, potentially reflecting aggregation, entrapment, or degradation. Moreover, when delivered via EDVs, only ~35% of the Cas9 protein remained intact, suggesting that misfolding or partial degradation during packaging can further compromise the editing activity. These results reinforce the need for delivery systems that preserve Cas9’s structural integrity and ensure effective nuclear localization [26].

Therefore, delivery systems must be carefully designed to maintain small and stable particle sizes during all stages of storage and administration.

Finally, aggregation can have different effects. On one hand, aggregates that are too large for endocytosis reduce cellular internalization, and on the other hand, moderate aggregation might enhance the local concentration or stability. Nevertheless, most evidence to date suggests that aggregation is unfavorable because it decreases the encapsulation efficiency, alters the particle geometry, and can impair nuclear uptake. For example, the electron microscopy of gold nanoparticles conjugated with Cas9 revealed clustered aggregates in some formulations, especially when the nitrilotriacetic acid coverage was low [74]. Strategies such as polyglutamic acid coating, or the use of pre-heated gRNA, have shown promising results regarding reducing aggregation effects [9,71]. Crucially, these findings suggest that not just the encapsulation amount, but also the encapsulation quality, including the aggregation state and release kinetics, should be optimized to unlock the full potential of Cas9-based therapeutics.

This functional loss due to aggregation or instability has also been observed in polymer-based systems. Chen et al. demonstrated that PEG–PLE-coated C14-PEI micelleplexes, designed for the co-delivery of Cas9 mRNA and sgRNA, achieved a narrow size distribution (~140 nm, PDI = 0.08), reduced zeta potential, and up to 69% editing efficiency in KRAS-mutant lung cancer cells [34]. In contrast, uncoated micelles showed greater polydispersity and surface charges, which correlated with an increased risk of aggregation or non-specific uptake [35]. Similarly, Abbasi et al. achieved ~20% gene editing in the brain using PEG–PLL polyplex micelles, highlighting the relevance of physicochemical control across different tissues [33]. These results confirm that the encapsulation efficiency is not solely defined by the loading capacity but also by the nanoparticle stability and physicochemical parameters, such as those optimized in polyplex micelles, that ensure intracellular functionality.

Furthermore, several studies have supported the strong correlation between the encapsulation efficiency and gene editing performance in both in vitro and in vivo systems. Im et al. reported that their LNPs delivering RNPs achieved up to a 45.2% indel frequency in *IL-10* gene editing, as confirmed by targeted deep sequencing in CT26 tumor cells [75]. Likewise, studies using virus-like particles demonstrated editing rates of 70–90% in human cells and confirmed functional gene knockout in preclinical models [76].

As previously discussed, the use of systems designed for lung and liver targeting, such as those reported by Wei et al., demonstrated that the LNP-mediated delivery of Cas9-RNPs enables robust gene editing (Table 1) with minimal off-target effects [22]. These findings exemplify the therapeutic relevance of non-viral platforms and help to explain the current shift away from viral vectors. Despite their historical predominance due to their high transduction efficiency, viral systems such as lentiviruses and AAVs face significant limitations, including immunogenicity, production constraints, and susceptibility to host restriction factors [46,49]. Consequently, non-viral strategies like lipid nanoparticles are gaining attention as safer and more flexible alternatives for in vivo genome editing.

**Table 2:** This table summarizes different Cas9 delivery platforms, focusing on the encapsulation efficiency, particle size, and gene editing outcomes. Note that not all studies in the bibliography reported precise encapsulation percentages or editing efficiencies.

Delivery System	Cas9 Encapsulation Efficiency	Particle Size (Hydrodynamic)	Gene Editing Efficiency/Therapeutic Outcome	Ref.
Exosomes (native)	~1% (low stochastic loading)	Not specified	Poor delivery, no editing data	[72]
Cas9 conjugated to a 12 nm gold nanoparticle	~45 Cas9 proteins per particle (~6%)	~23 ± 5 nm	Comparable to electroporation in reported assays	[74]
LNPs	Not reported numerically; varied with RNP ratio	~100–200 nm (est.)	Up to 45.2% indels in *IL-10* gene	[75]
LNPs	Not quantified	Not specified	~3–3.5% HDR integration; 80% restoration of cystic fibrosis transmembrane conductance regulator chloride channel function	[77]
LNPs	Not reported (mRNA and sgRNA co-delivered)	81–99 nm (PDI of 0.19–0.22)	39.1% editing in liver; phenylalanine levels normalized within 48 h	[78]
LNPs	Not specified	Not reported	Exon skipping of ~90% in APP; ~70% reduction in Aβ42 in vivo	[79]
LNPs	Up to 98% depending on formulation	112–176 nm (PDI of ~0.10–0.17)	37% in liver, 19% in lung (in vivo); >90% in vitro (NPCs, HEK)	[32]
Virus-like particles	Not specified, but functional loading confirmed	~100–200 nm	Knockout efficiency of 70–90% in vitro and 60–70% in primary human T cells	[76]
Gold nanoparticle aggregates	Surface clustering observed (variable density)	>40 nm in aggregated states	Decreased nuclear entry and editing when aggregation was not controlled	[74]
PEG–PLL polyplex micelles	Co-encapsulation of Cas9 mRNA and sgRNA confirmed; no quantitative encapsulation efficiency reported	~30–35 nm	~20% editing in neurons, astrocytes, and microglia after brain injection	[33]
C14–PEI micelleplexes	Encapsulation stability confirmed; some aggregation	~140 nm	60% (T7EI) adn 48.5% (ddPCR) in KRAS-mutant lung cells	[34]
PEG–PLE/C14–PEI micelleplexes	High stability, PEG shield reduced non-specific interactions	~120 nm	Up to 69% indels; improved lung delivery	[35]
EDVs	~35% of Cas9 remained intact post-packaging	~120–140 nm (estimated)	Accelerated editing kinetics	[26]

## 5. Clinical Translation Barriers: Manufacturing, Scalability, and Regulatory Considerations

Despite notable advances in CRISPR/Cas9 delivery platforms, their transition into clinical practice remains limited by challenges in their manufacturing scalability, regulatory oversight, and reproducibility.

For example, strict control must be maintained over the physicochemical characteristics (such as the particle size, encapsulation efficiency, and batch uniformity) of lipid nanoparticles (LNPs) and other non-viral carriers during upscaling, which is technically demanding and cost-intensive. As outlined by the FDA, even minor changes in the manufacturing process of gene therapy products require comprehensive comparability data to ensure that safety and efficacy remain consistent. Their guidance emphasizes the need to validate analytical methods to monitor the potency, identity, and purity across different production batches [77].

A major regulatory requirement for the clinical translation of gene editing therapies is the robust characterization of delivery systems. According to the European Medicines Agency (EMA), delivery platforms (whether viral or non-viral) must be well defined in terms of their vector composition, stability, and biological activity and must demonstrate reproducibility across manufacturing scales [80]. This is particularly critical for complex nanomaterials, which are highly sensitive to physicochemical variations and subtle batch-to-batch differences. The 2025 EMA Horizon Scanning Report on nanotechnology-based medicinal products emphasizes this issue, calling for advanced analytical strategies to monitor the particle size, surface properties, and encapsulated biomolecule integrity [81]. In parallel, regulatory guidelines highlight the need for long-term patient follow-ups and environmental risk assessments for in vivo applications, especially in the case of systemic genome editing [82].

In addition to these technical and regulatory hurdles, financial and infrastructural limitations remain significant barriers to widespread clinical implementation. Recent reports underscore the challenges faced by healthcare systems in sustaining access to high-cost gene therapies, particularly those involving one-time or short-term administration with durable clinical benefits. The lack of harmonized reimbursement schemes, the high cost of production, and the limited infrastructure for post-treatment monitoring have been repeatedly cited as critical bottlenecks. A recent scoping review by Ossandon et al. (2024) analyzed ten global financing strategies for gene therapies and concluded that although models like outcome-based payments, amortization, and subscription pricing are promising, their implementation is still hindered by administrative complexity, data collection limitations, and regulatory fragmentation [83].

Taken together, these factors highlight the importance of not only improving the delivery efficiency but also ensuring manufacturing consistency, regulatory compliance, and the equitable deployment of CRISPR/Cas9-based therapies in clinical settings.

Additionally, functional barriers such as Cas9 aggregation or degradation during formulation can critically impact the therapeutic efficacy and batch reproducibility. For instance, Karp et al. reported the partial degradation of Cas9 in enveloped delivery systems and cytoplasmic retention after electroporation, both of which indicate a potential loss of activity [26]. These findings suggest that, beyond the loading capacity, maintaining molecular stability is essential to ensure consistent outcomes across production lots.

Equally important are strategies to overcome the organ tropism limitations of conventional LNPs, which typically accumulate in the liver. SORT systems provide a promising solution by fine-tuning the lipid composition to achieve tissue-specific delivery. Wang et al. and Su et al. demonstrated that SORT LNPs enable reproducible targeting to the lung and spleen, even after systemic administration, offering a scalable route to extrahepatic gene editing [59,60]. However, these advanced formulations also introduce new manufacturing and regulatory complexities, as small changes in lipid ratios can shift the biodistribution profiles, potentially requiring additional safety and comparability testing under current FDA and EMA frameworks.

The systemic delivery of base editors, as demonstrated by Bzhilyanskaya et al., confirms the feasibility of achieving high editing efficiency in clinically relevant targets such as hematopoietic stem cells [67]. However, immune considerations remain essential; Charlesworth et al. identified pre-existing adaptive immunity to Cas9 in human serum, and Toral et al. found tissue-specific immunogenicity differences, with immune-privileged sites like the eye potentially offering safer locations for CRISPR therapies [11,12].

## 6. Conclusions and Future Perspectives

The CRISPR/Cas9 technique has completely revolutionized genome editing’s scope since its discovery, providing an accurate and efficient method for DNA modification and opening alternative approaches to numerous illnesses due to its undoubtedly high therapeutic potential.

The future of gene therapy using CRISPR/Cas9 is based on the development of advanced and specific delivery systems, as well as on improving the precision of gene editing. Recent advances in nanotechnology, particularly in the use of LNPs, polymeric carriers, and other non-viral vectors, have significantly enhanced the safety and efficiency of CRISPR/Cas9 delivery systems. These systems not only improve the cellular uptake but also provide enhanced cargo protection, reducing the risk of off-target effects and enabling tissue-specific editing in organs, representing a critical step towards clinical translation [24,45].

Moreover, recent evidence has shown that editing outcomes in vivo are closely related to the quality of Cas9 encapsulation and its aggregation state. For example, systems achieving over 70% gene knockout in human cells or restoring up to 80% CFTR activity in animal models support the idea that both functional delivery and the therapeutic efficiency depend on optimal Cas9 loading and release [76,84].

Further investigation will provide new approaches, reducing side effects and improving CRISPR/Cas9’s safety. The exploration of novel Cas proteins, such as Cas12, can improve specificity and reduce immunogenicity in genome editing. Therefore, this enzyme can expand gene therapy approaches, providing more precise targeting capabilities and the potential to address a broader range of genetic disorders [85]. Moreover, optimizing the Cas9/gRNA ratio to improve RNP formation may enhance the editing efficiency [86]. In addition, structural studies using techniques like high-speed atomic force microscopy and enhanced molecular simulations have provided deeper insights into Cas9’s mechanistic features, clearing the path for highly precise genome editing tool development [87,88].

As highlighted in this review, CRISPR/Cas9 delivery systems, especially non-viral delivery vehicles including LNPs, have demonstrated promising results in improving gene editing’s safety, efficiency, and specificity. These systems reduce the risks associated with viral vectors, such as immunogenicity and off-target genomic integration, while allowing for precise editing in a wide range of tissues. However, Cas9 aggregation could compromise the internalization in cells and encapsulation in delivery systems and therefore reduce the genome editing efficiency; therefore, a deeper understanding of the molecular mechanisms driving aggregation is essential to develop alternative strategies.

One of the most significant barriers in systemic CRISPR/Cas9 delivery remains the non-specific biodistribution of nanoparticle carriers, especially LNPs, which tend to accumulate in hepatic tissue. To overcome this, SORT approaches have been developed to retune lipid compositions and redirect nanoparticle tropism toward the desired organs. Wang et al. demonstrated that using cationic (DOTAP) or anionic (18PA) lipids as a fifth component enabled the selective delivery of CRISPR/Cas9 cargo to the lung or spleen, respectively, following intravenous injection [59]. Likewise, Su et al. showed that cholesterol-free lipid formulations could significantly increase the pulmonary accumulation of mRNA and reduce liver exposure, suggesting a route toward safe, efficient in vivo editing in non-hepatic tissues [60]. These SORT LNP platforms exemplify the growing potential of strategic lipid engineering to achieve organ-specific CRISPR/Cas9 delivery, a crucial advancement for future clinical translation.

Given its potential implications, future research should focus on analyzing Cas9 aggregation across different delivery methods. Techniques such as DLS, FCS, and size exclusion chromatography could be employed to quantify aggregation levels and evaluate their effects on the gene editing efficiency. In addition, considering in vivo applications, it is necessary to explore whether Cas9 aggregates show preferential accumulation in certain tissues or induce unwanted immune reactions. Addressing these questions will be essential to optimize CRISPR/Cas9 delivery strategies and enhance the technique’s therapeutic applications.

In parallel, efforts to correlate encapsulation parameters, such as the Cas9 loading per particle or encapsulation efficiency, with the genome editing performance, will provide a more predictive framework for clinical translation. Quantifying how much Cas9 remains encapsulated, and under which physicochemical conditions delivery improves, should become standard practice in therapeutic development. Recent clinical and preclinical studies have demonstrated the growing potential of CRISPR-based therapeutics delivered via non-viral platforms. Chen et al. reported efficient in vivo genome editing in the liver and lung using LNP-formulated Cas9 RNPs [32]. In parallel, base editors have shown therapeutic efficacy across multiple contexts: Bzhilyanskaya et al. achieved PAMless base editing in hematopoietic stem cells; Brooks et al. corrected a metabolic mutation in a humanized mouse model of phenylketonuria; and Miskalis et al. demonstrated exon skipping in the brain using a near-PAMless base editing platform [78,79]. The outcomes and delivery parameters of these studies are further detailed in Table 1 and Table 2, illustrating the translational impact of recent LNP-based approaches. These findings reinforce the need for continued innovation in precise, efficient, and scalable genome editing strategies.

The clinical success of CASGEVY^TM^ demonstrates the therapeutic viability of CRISPR/Cas9 systems using non-viral delivery [9,10]. However, its reliance on ex vivo electroporation and stem cell manipulation confines its use to hematologic conditions and specialized facilities. These limitations emphasize the urgent need for next-generation delivery platforms, such as lipid-based platforms or polymeric nanoparticles, that enable precise, systemic in vivo editing across a broader range of diseases and tissues.

Despite technological improvements, ethical concerns surrounding CRISPR/Cas9’s application remain present. Germline editing, in particular, raises profound moral and social questions due to its heritable nature, which could lead to unintended long-term implications of genetic modifications. As a result, establishing robust regulatory frameworks is essential to ensure a balance between innovation and safety, thus avoiding potential risks and unintended genomic alterations. Collaborative efforts among scientists, politicians, and society will be critical to establish guidelines that balance innovation with safety.

Despite these advances, several research gaps remain underexplored. Comparative studies evaluating the performance of viral versus non-viral systems, particularly in sensitive cell types such as hematopoietic stem cells and T lymphocytes, are still limited. Moreover, there is currently no consensus on standardized metrics for quantifying the encapsulation efficiency, stability, or intracellular release across different delivery platforms, making it difficult to benchmark or compare outcomes. Lastly, although strategies like base editing and prime editing offer improved precision, little is known about their tissue-specific delivery kinetics, biodistribution profiles, or long-term functional impact following systemic administration. Addressing these questions will be essential for the rational design and clinical implementation of next-generation gene editing systems.

Ultimately, realizing the full clinical potential of CRISPR requires a multi-perspective approach that addresses technical, ethical, and social challenges, opening the door to safer, more effective, and universally accessible gene editing solutions.

## Abbreviations

The following abbreviations are used in this manuscript:

AAVs	Adeno-associated viruses
ABE	Adenine Base Editor
ADGNs	Artificially Designed Grafting Nanoparticles
ATMPs	Advanced Therapy Medicinal Products
Cas9	CRISPR-associated protein 9
CDK11	Cyclin-dependent kinase 11
CFTR	Cystic fibrosis transmembrane conductance regulator
CPP	Cell-Penetrating Peptide
CRISPR	Clustered regulatory interspaced short palindromic repeats
ddPCR	Droplet digital PCR
DLS	Dynamic light scattering
DOPE	1,2-dioleoyl-sn-glycero-3-phosphorylethanolamine
EDVs	Mini enveloped delivery vehicles
EMA	European Medicines Agency
FCS	Fluorescence correlation spectroscopy
FDA	Food and Drug Administration
gRNA	Guide RNA
HDR	Homology-Directed Repair
iPSCs	Induced pluripotent stem cells
LNPs	Lipid nanoparticles
mRNA	Messenger RNA
NPCs	Neural Progenitor Cells
PBS	Phosphate-Buffered Saline
PDI	Polydispersity Index
pDNA	Plasmid DNA
PEG	Polyethylene glycol
PEI	Polyethylenimine
PMs	Polyplex micelles
RNP	Ribonucleoprotein
SORT	Selective Organ Targeting
ZIFs	Zeolitic imidazolate frameworks
